# Pediatric Viper Envenomation Complicated by Compartment Syndrome

**DOI:** 10.7759/cureus.101295

**Published:** 2026-01-11

**Authors:** Hosam M Elghadban, Deep Parkash, Al Majd A Al Yazidi

**Affiliations:** 1 General Surgery, Ibra Hospital, Ibra, OMN; 2 General Surgery, Ibra Hospital, Ministry of Health, Ibra, OMN; 3 Surgery, Ibra Hospital, Ibra, OMN

**Keywords:** antivenom therapy, emergent fasciotomy, envenomation, foot compartment syndrome, snake-bite

## Abstract

Snake envenomations can cause both severe systemic and local complications. Acute compartment syndrome (ACS) is a rare but severe limb-threatening complication. Due to limited communicability and anticipated envenomation-related swelling, it is often subtle and difficult to diagnose in pediatric populations.

A previously healthy three-year-old female presented one hour after sustaining a bite to the dorsum of her left foot by a saw-scaled viper (Echinus carinatus). A common viperidae in the region of Oman, it is responsible for a significant amount of annual envenomations leading to coagulopathy.

Upon admission, the patient developed rapid swelling of the left foot and profound coagulopathy. The antivenom therapy (EQUINE Serum, Ministry of National Guard, Health Affairs, KSA) was initiated within 90 minutes of envenomation. Serial Doppler ultrasounds were performed with a change from triphasic to monophasic flow and ultimately, barely detectable dorsalis pedis arterial waveforms. Capillary refill time was delayed at 4-5 seconds (normal <2 s).

Due to clinical concern for acute compartment syndrome (ACS), an emergent fasciotomy was performed within three hours of admission. Intraoperatively, arterial perfusion improved significantly. Postoperatively, the wounds were left open with regular dressing changes. The patient was found to have healing through secondary intention.

This case highlights a potential yet rare complication of envenomation-induced compartment syndrome in a pediatric patient. Early recognition, surgical decompression, and careful postoperative wound care can prevent limb loss and preserve function.

## Introduction

Snake envenomation in children can cause severe systemic and local complications. Acute compartment syndrome (ACS) is a rare but limb-threatening outcome, often difficult to diagnose in pediatric patients due to limited clinical communication and overlapping features with envenomation-related swelling [1].

Snakebite envenomation remains a significant public health concern worldwide. The World Health Organization (WHO) classifies it as a neglected tropical disease, a designation reaffirmed in 2017. This classification is especially relevant to the Middle East and Oman due to regional factors, such as limited access to specialized antivenom in rural areas, delayed presentation, often resulting from dependence on traditional medicine practices, and under-recognition of ACS as a potential complication. Recognizing these regional challenges can help us improve patient outcomes [2,3].

Due to their smaller body mass, children are particularly vulnerable to severe complications from a higher venom-to-bodyweight ratio. Envenomation caused by the saw-scaled viper (Echis carinatus) features a venom profile that differs significantly from that of North American crotalids. Regional vipers, such as Echis and Daboia, induce severe coagulopathy, similar to that observed in timber rattlesnakes, necessitating aggressive antivenom treatment. Usually, it causes coagulopathy, bleeding, and local swelling. The occurrence of ACS is rare, estimated to occur in approximately 0.5% to 3% of snakebite envenomation cases. Diagnosing ACS in children is particularly difficult because pain and paresthesia, which are key signs in adults, are difficult to ascertain or communicate with young patients [4].

The diagnostic criteria for ACS include a compartment pressure of 45 mmHg (diagnostic), a delta pressure of 13 mmHg (diagnostic for ACS, threshold < 30), a serial Doppler showing progression from triphasic to monophasic to barely detectable, a capillary refill of 5 seconds (normal < 2 s), a temperature differential of 2 °C cooler, and circumferential measurements of an 8 cm increase at the ankle and 12 cm at the calf. Additionally, it is often challenging to distinguish between severe venom-induced swelling and true compartment syndrome. Recognizing these vulnerabilities can guide more attentive care [5].

Here, we report a pediatric case of snakebite envenomation complicated by ACS that required fasciotomy, with emphasis on the clinical course, management decisions, and outcomes. This case aims to provide insights into early recognition, diagnostic challenges, and treatment approaches for similar cases in clinical practice.

## Case presentation

A previously healthy 3-year-old female presented to the emergency department at a tertiary care hospital in Muscat, Oman, in July 2024 (peak summer season, temperature 42 °C), approximately one hour (T1h) after sustaining a saw-scaled viper (Echis carinatus) bite (T0) to the dorsum of her left foot between the fourth and fifth metatarsals. The envenomation occurred while the individual was playing in the family's backyard garden at dusk (T0), a period of peak activity for this nocturnal species.

On presentation, she was alert and interactive, with no systemic complaints, including shortness of breath, chest pain, or neurological deficits. Vital signs included temperature 37.2 °C, heart rate 118 bpm, respiratory rate 30/min, blood pressure 95/58 mmHg (normal for age), and SpO2 98% on room air. Examination revealed two puncture marks 8 mm apart on the dorsum of the left foot with persistent oozing of blood (approximately 5 mL over 10 minutes, not controlled by pressure). The foot demonstrated moderate pitting, 4 mm deep, disappearing in ~10-15 seconds (2+), and extending to the ankle, with a circumference 8 cm greater than the contralateral foot. The skin was warm, erythematous, and tense to palpation. No odor was detected. Dorsalis pedis and posterior tibial pulses were normal, easily palpable (2+), and palpable bilaterally at this time. The child was crying inconsolably, making pain assessment difficult beyond localization to the affected foot.

**Figure 1:**
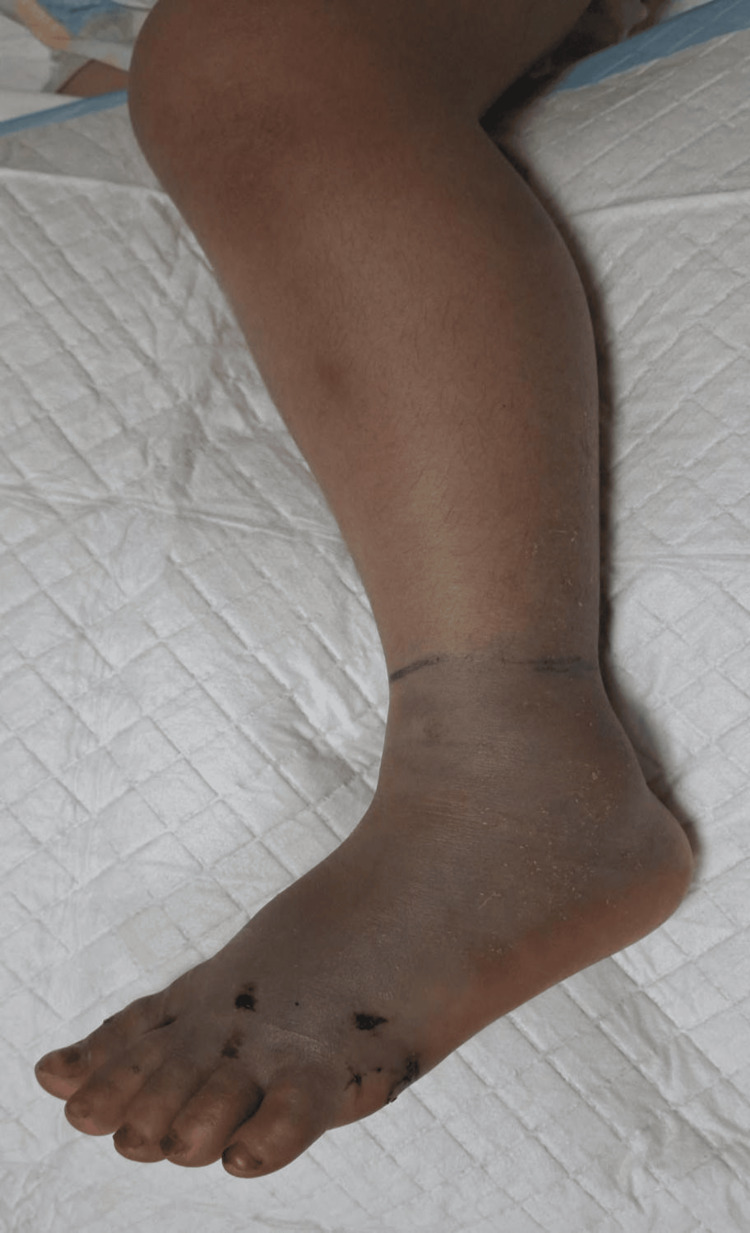
Pre-fasciotomy image with color change and swollen left foot The dark line indicates the proximal extent of color changes at the time of the photograph (T+3h), extending to the distal calf. This was used for follow-up on the improvement of the condition after surgical intervention.

Within hours of admission, swelling spread beyond the ankle and later extended above the knee. The discoloration was cyanotic (blue-purple) rather than ecchymotic (yellow-green bruising), localized distally in the toes and forefoot rather than at the bite site, and was accompanied by coolness to touch (foot temperature 2 °C cooler than the contralateral foot), and passive toe extension elicited severe pain response, suggesting ischemia rather than coagulopathic bleeding, as in Figure 1. Pulses were initially palpable at presentation (T+1h), became non-palpable but Doppler-detectable with normal waveforms at T+2h, and at T+3h, were clinically non-palpable but remained detectable by Doppler, with diminished waveforms (monophasic vs. initially triphasic), suggesting compromised arterial flow beyond simple venous congestion from edema. By T+3.5h, even Doppler signals were barely detectable in the dorsalis pedis. The capillary refill was delayed at 4-5 seconds (normal <2 s). The limb was tense and extremely tender, and passive toe movement was painful. The initial coagulation profile showed severe hematotoxic envenomation: PT and thrombin time were undetectable, aPTT was >120 s, and fibrinogen levels were reported as below the assay’s measurable limit. CBC showed hemoglobin 10.1 g/dL, WBC 11.8 × 10³/μL, and platelets 165 × 10³/μL. Hypokalemia (2.8 mmol/L) was detected. Over the next 24 hours, coagulation parameters gradually improved with repeated doses of antivenom, eventually normalizing.

Following our institutional severe envenomation protocol (adapted from WHO guidelines 2016), which defines severe envenomation as coagulopathy with an international normalized ratio (INR) of >3, fibrinogen < 100 mg/dL, or evidence of systemic toxicity. Polyvalent snake antivenom (EQUINE Serum, Ministry of National Guard, Health Affairs, KSA, containing equine-derived immunoglobulins against Gitis arietans venom, Cerastes cerastes venom, Echis carinatus venom, Echis coloratus venom, Naja haje venom, Watterinnesia aegyptia venom, each 1 ml neutralizes ≥25 LD50; manufactured by the National Antivenom and Vaccine Production Center). Initial dose: 10 vials (each 10 mL) diluted in 200 mL normal saline, infused over 1 hour starting at T+1.5 h (90 minutes post-bite). The patient was monitored for anaphylaxis (none observed). Repeat dosing was 6 vials at T+3h and 4 vials at T+6h based on persistent coagulopathy. This was a total of 20 vials over 12 hours until INR < 1.5 and fibrinogen > 100 mg/dL were achieved. Analgesia included morphine sulfate, continuous IV infusion at 10 mcg/kg/hr, initiated at T+2h and continued for 72 hours post-fasciotomy, then weaned over 24 hours, Paracetamol (acetaminophen): 15 mg/kg/dose IV (450 mg/dose) every 6 hours (total 1,800 mg/24h, well within the safe limit of 60 mg/kg/24h, started on presentation and continued for 7 days and Tramadol, 1 mg/kg IV as needed for breakthrough pain, maximum 4 doses/24h (used twice on day 1, once on day 2). Pain scores (FLACC (Face, Legs, Activity, Cry, Consolability) scale for non-verbal assessment) were 8/10 pre-fasciotomy, 4/10 post-fasciotomy, and 2/10 by day 3. This standardized reporting demonstrates appropriate weight-based dosing and safe limits. Intravenous fluids with potassium supplementation were given for hypokalemia. Limb elevation and frequent pulse checks were maintained.

Despite optimal medical therapy, including high-dose antivenom, swelling worsened, extending above the knee with greenish discoloration and tense compartments. Clinical suspicion of compartment syndrome was raised due to absent palpable pulses, severe tenderness, and compromised perfusion indicated by a capillary refill of 5 seconds (normal <2s); a temperature differential of 2 °C cooler; and circumferential measurements of a 6 cm increase at the ankle and 10 cm at the calf. Serial Doppler ultrasound using handheld continuous-wave Doppler ultrasound (8 MHz probe, Huntleigh Dopplex® DMX, Huntleigh Healthcare, UK) for pulse detection and waveform analysis showed a progressively diminished arterial flow at the dorsalis pedis and posterior tibial arteries. Bedside pressure measurement of the anterior compartment was not performed due to the unavailability of the device. A multidisciplinary discussion including general surgery, orthopedic surgery, emergency medicine, and the pediatric intensive care team. A consensus was reached that clinical and objective findings met the criteria for ACS requiring emergent fasciotomy; emergency fasciotomy was planned.

**Figure 2:**
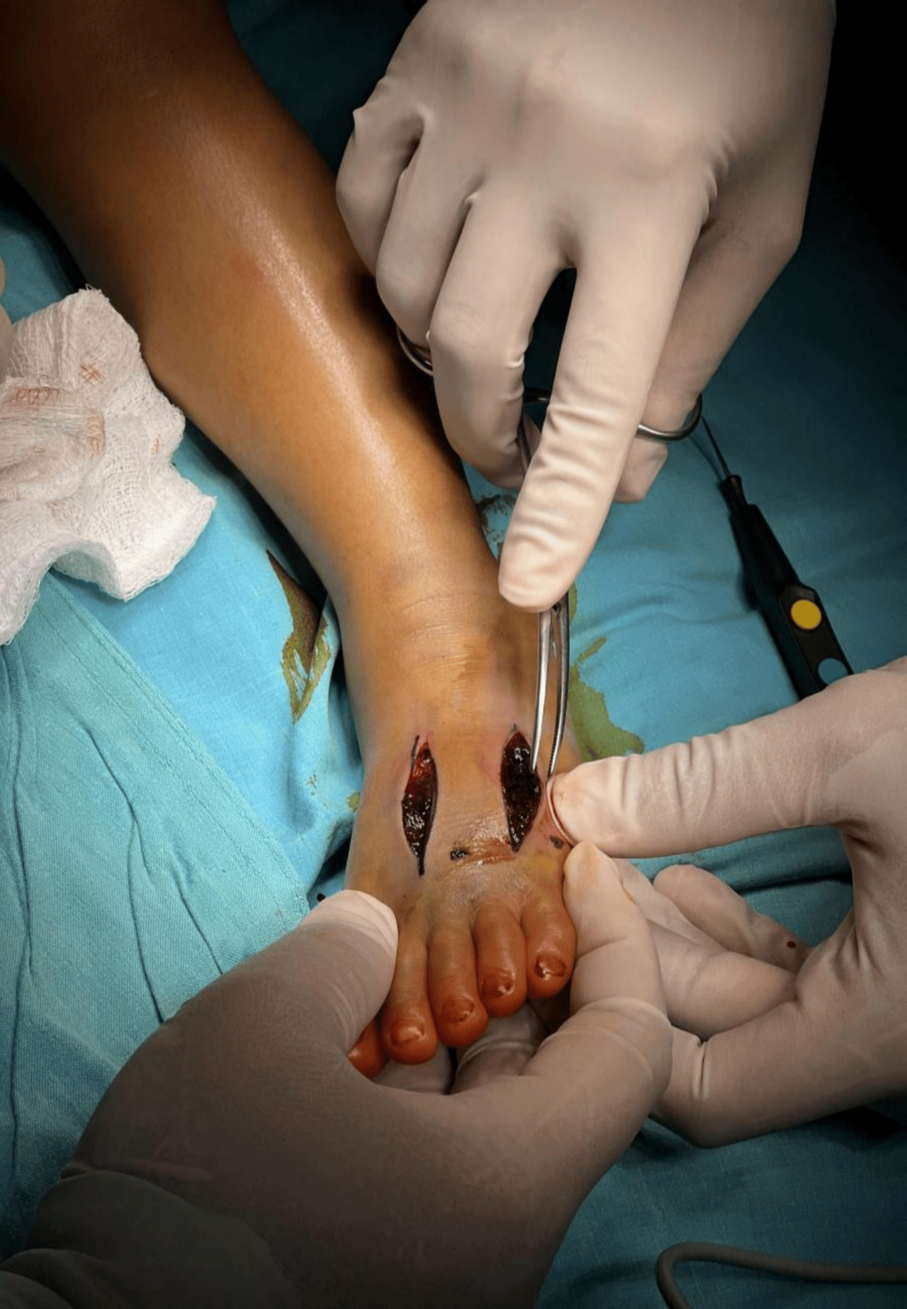
Intraoperative image demonstrates significant tension and dark colour of the muscle Note the cyanotic muscle tissue in the anterior compartment (center) compared to the healthier pink muscle laterally. The dark material is a combination of edema fluid and blood from coagulopathy, not necrotic tissue. Muscle contractility was preserved upon stimulation, confirming viability. Tissue tension is evidenced by the immediate bulging of muscle through the fasciotomy incisions upon release.

**Figure 3:**
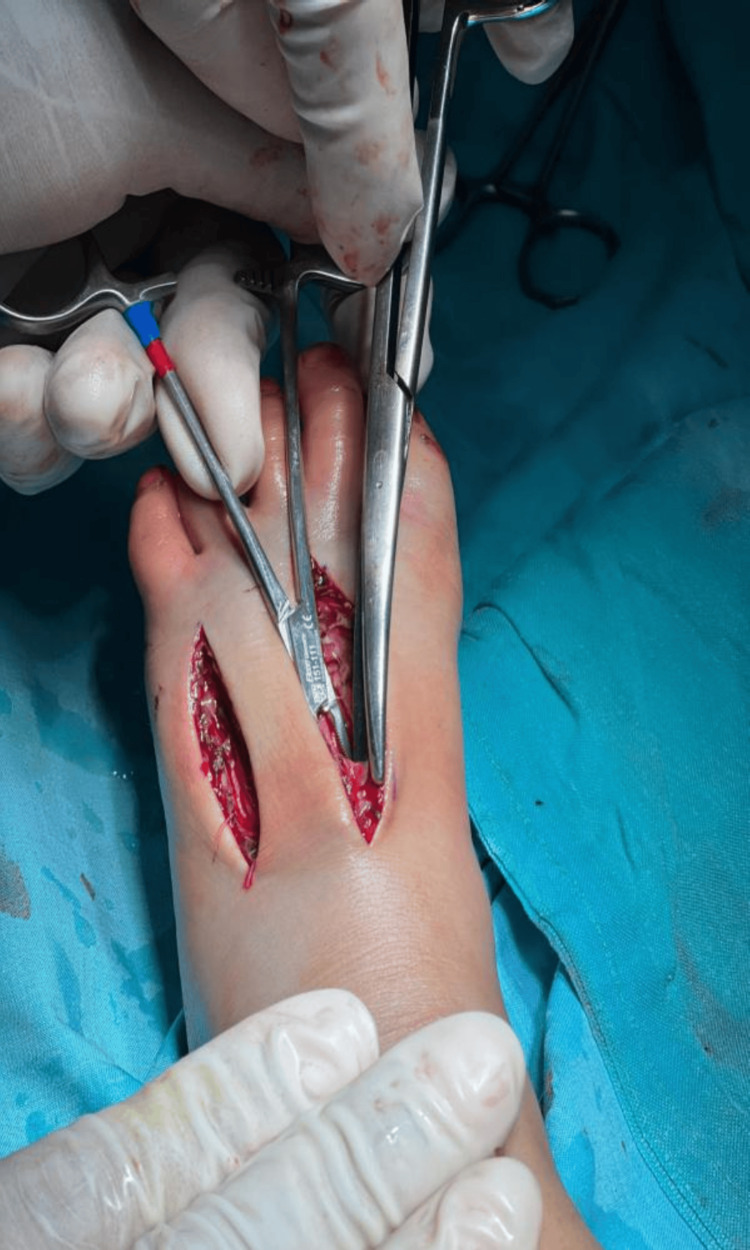
Intraoperative image showing the fasciotomy incisions with immediate release of tense compartments The fascial compartments were released and the flexor retinaculum was incised.

Fasciotomy was performed under general anesthesia. Three incisions were made: a 6 cm incision at the second metatarsal space, a 5 cm incision at the fourth metatarsal space, and a 4 cm incision along the medial aspect of the foot. Intraoperative findings included severe edema, cyanotic discoloration (not bruising/ecchymosis) of the skin of the ankle indicating ischemia, distinct from the localized ecchymosis at the fang puncture sites, and tense calf compartments, as shown in Figure 2. Upon decompression that was extended up to the flexor retinaculum, perfusion improved, and the toes regained warmth and color, as shown in Figure 3. Hemostasis was secured, and the wounds were left open with paraffin gauze and wet dressings. No drain was placed.

Tetanus toxoid booster was administered (last dose was 18 months prior; amoxicillin-clavulanate 30 mg/kg/dose IV every 8 hours (900 mg/dose for a 30 kg patient) and post-fasciotomy (T+4h) was initiated for surgical wound prophylaxis and continued for 48 hours. On postoperative day 2, wound inspection revealed increased erythema at the medial incision site (2 cm surrounding margin, warmth, no purulent discharge). Wound culture was obtained. Ceftriaxone (50 mg/kg/dose IV once daily) + Cloxacillin (50 mg/kg/dose IV every 6 hours) were initiated on postoperative day 2 for broader gram-positive coverage (particularly anti-staphylococcal given skin flora concern) while awaiting culture results. The doses were continued for seven days (five additional days after the switch). The culture result demonstrated light growth of Staphylococcus epidermidis (skin contaminant vs. colonization), sensitive to cloxacillin. The clinical outcome was that erythema resolved by day 4, and the wounds were clean with healthy granulation tissue. Table 1 shows the timeline.

**Table 1:** Timeline of the case progression T = time

Time	Event
T0	Bite time
T+1h	ED arrival
T+1.5h	Antivenom started
T+3h	Swelling in the knee
T+4h	Fasciotomy performed
T+12h	Coagulation normalized

**Figure 4:**
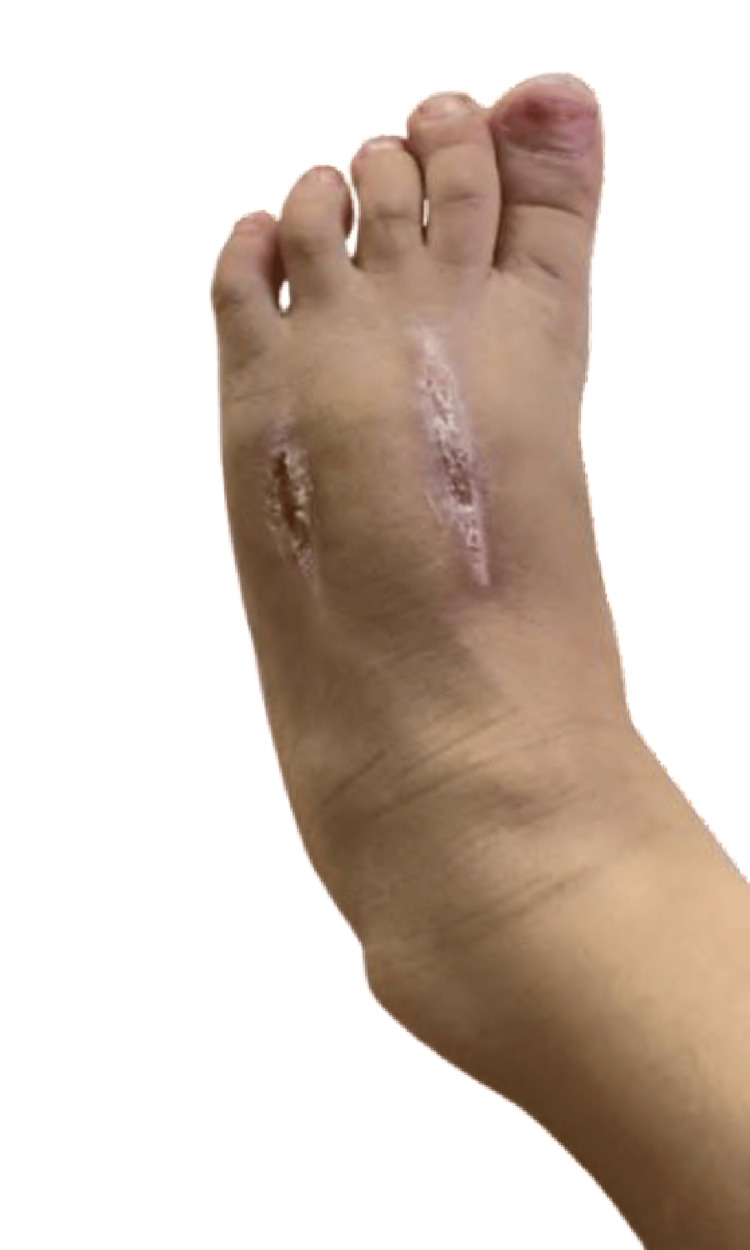
Complete healing at three weeks post-fasciotomy. Wounds healed by secondary intention without the need for skin grafting or delayed primary closure. Note well-formed scar tissue with preserved foot architecture and function. The patient demonstrated full weight-bearing and normal gait.

Following fasciotomy, Doppler signals returned to biphasic waveforms within 30 minutes, capillary refill improved to 2 seconds, and toe temperature normalized within 2 hours, objectively confirming restoration of perfusion. Separately, coagulopathy normalized over 18 hours post-initial antivenom (not related to fasciotomy), with INR reaching 1.4, APTT 38 seconds, and fibrinogen 120 mg/dL by T+20h, attributed to antivenom effect and hepatic synthesis recovery. The patient remained hospitalized for 10 days total: 3 days in the PICU for hemodynamic monitoring and coagulopathy management, and 7 days in the pediatric surgery ward for wound care. She was discharged on day 10 with outpatient wound clinic follow-up. She remained afebrile and hemodynamically stable, with dorsalis pedis and posterior tibial pulses that were both palpable (2+) bilaterally, compared to non-palpable on the affected side preoperatively, and warm toes. Antibiotics were continued, and analgesia was maintained. After one week, wounds were clean, swelling had decreased markedly, and granulation tissue appeared. By the third week, the wounds remained dry and clean, healing well without the need for sutures. The fasciotomy sites healed by secondary intention with regular dressings, and the patient retained normal limb function and was able to walk (Figure 4).

## Discussion

The WHO has classified snakebite envenomation as a neglected tropical disease, a classification that is particularly relevant to our region (Middle East/Oman) due to limited access to specialized antivenom in rural areas, delayed presentation due to traditional medicine practices, and under-recognition of ACS as a complication. In this case, the patient presented within 1 hour, which is relatively prompt, but regional data shows 40% present >6 hours post-bite.

Snake-scaled viper (Echis carinatus) envenomation can result in a broad spectrum of complications, ranging from mild local swelling to life-threatening systemic effects, owing to its hemotoxic venom profile, which differs significantly from that of New World crotalids. Regional viper species (Echis, Daboia) cause severe coagulopathy, similar to that observed in timber rattlesnakes, requiring aggressive antivenom protocols. The polyvalent antivenom used in our region follows a different dosing regimen than CroFab/ANAVIP due to different venom potency and antibody titers. Our protocol is consistent with WHO recommendations for Echis envenomation (10-20 vials for severe cases). While tissue swelling is common, progression to ACS is rare. Venom-induced vascular permeability and tissue necrosis lead to rapid fluid accumulation in fascial compartments [4]. Increased intracompartmental pressure compromises microvascular perfusion, leading to ischemia and, if untreated, potential tissue loss. Clinicians should monitor for signs such as increased swelling, tense compartments, and decreased perfusion, as evidenced by pale or cool skin. Prompt recognition and intervention based on these cues may prevent irreversible damage [4].

In children, classic symptoms of ACS--pain out of proportion, paresthesia, and paralysis--may be challenging to assess. The diagnostic criteria met in this case include several clinical and vascular signs consistent with ACS. These encompass a tense compartment, pain with passive stretch, and paresthesia or pain that is disproportionate to the injury, as assessed by behavioral responses in this pediatric patient. Additionally, vascular compromise was evidenced by diminished Doppler signals, delayed capillary refill, and temperature differential. The progression of these signs persisted despite antivenom administration. These criteria, originally established by Whitesides et al. and adapted for pediatric patients, serve to confirm the diagnosis of ACS rather than vascular injury due to envenomation alone [6,7].

The utility of fasciotomy in the setting of snake envenomation is widely debated, with most literature focusing on crotalid (pit viper) bites. For viper envenomations (including those caused by Echis species), the evidence is limited but suggestive. Some studies caution against it, citing risks of infection and worsened tissue necrosis. However, in cases with clear evidence of vascular compromise, surgical decompression is essential [8,9]. Clinical suspicion of compartment syndrome was raised due to absent palpable pulses, severe tenderness, and compromised perfusion indicated by capillary refill of 5 seconds (normal <2s); a temperature differential of 2 °C cooler; and circumferential measurements of a 6 cm increase at the ankle and 10 cm at the calf. Serial Doppler ultrasound showed a progressively diminished arterial flow at the dorsalis pedis and posterior tibial arteries. Bedside pressure measurement of the anterior compartment was not performed because the device was unavailable. Fasciotomy was initially focused on the foot compartments. Intraoperatively, upon release of the foot compartments and flexor retinaculum, calf swelling decreased, and leg compartment palpation revealed adequate compliance; thus, leg fasciotomy was deemed unnecessary. Fasciotomy restored perfusion, as evidenced by objective improvements in Doppler signals and capillary refill, rather than by preventing necrosis.

Institutional protocol addresses secondary infection after fasciotomy (not preoperatively for the bite itself). Open wound management planned. Antibiotics were initiated post-fasciotomy, not at presentation. Tetanus toxoid booster administered (last dose 18 months prior, up-to-date per schedule).

Leaving fasciotomy wounds open is standard. However, the closure strategy varies. In this case, conservative management with dressings and limb elevation led to healing by secondary intention, resulting in good cosmetic and functional outcomes. This case demonstrates that delayed closure or grafting is not always necessary, particularly in pediatric patients with strong healing potential [6,9].

## Conclusions

This case highlights a rare, yet potentially serious, complication associated with envenomation-induced compartment syndrome in a pediatric patient, emphasizing the critical importance of prompt recognition and a multidisciplinary approach. While antivenom therapy remains the cornerstone of treatment for snakebite envenomation, surgical decompression through fasciotomy is rarely indicated and typically reserved for cases where objective evidence of acute compartment syndrome persists despite adequate antivenom administration. In this instance, fasciotomy was performed only after the administration of 20 vials of antivenom; serial Doppler ultrasound examinations revealed progressively diminished arterial flow in the dorsalis pedis and posterior tibial arteries; and vascular compromise continued despite ongoing therapy. It is imperative to note that fasciotomy is not a routine intervention and should be contemplated solely when objective diagnostic criteria for ACS are met, despite optimal medical management.

Given the rarity and diagnostic challenges of envenomation-induced ACS, we propose a multicenter prospective registry to capture the incidence, risk factors, diagnostic criteria, and outcomes. Such data would inform evidence-based guidelines for surgical intervention thresholds in this controversial clinical scenario.
